# *In vivo* Anticancer Activities of Benzophenone Semicarbazone against Ehrlich Ascites Carcinoma Cells in Swiss Albino Mice

**DOI:** 10.7497/j.issn.2095-3941.2012.04.004

**Published:** 2012-12

**Authors:** Khairul Islam, Shaikh M Mohsin Ali, Mele Jesmin, Jahan Ara Khanam

**Affiliations:** 1 Department of Applied Chemistry and Chemical Engineering, University of Rajshahi, Rajshahi 6205, Bangladesh; 2 Department of Biochemistry and Molecular Biology, University of Rajshahi, Rajshahi 6205, Bangladesh

**Keywords:** anticancer activities, EAC cell, benzophenone semicarbazone

## Abstract

**Objective:**

Benzophenone semicarbazone (BSC) was synthesized and characterized to identify compounds with anticancer activities.

**Methods:**

Anticancer activities were studied against Ehrlich Ascites Carcinoma (EAC) cells in Swiss albino mice by monitoring parameters such as tumor weight measurement, survival time of tumor bearing mice, tumor cell growth inhibition, and so on. Some hematological parameters, such as red blood cells, white blood cells, and hemoglobin content, were also measured.

**Results:**

The results showed that BSC has a positive effect against EAC cells. An assessment was conducted by comparing these results with those obtained using the standard drug bleomycin.

**Conclusions:**

The BSC compound can be considered as a potent anticancer agent.

## Introduction

Many natural and synthetic compounds are capable of affecting selectively specific organs and tissues within a biological system. Among such compounds, Schiff bases are well known for their importance in biological activities as antimicrobial^[^^1^^-^^3^^]^, anti-inflammatory^[^^4^^,^^5^^]^, analgesic^[^^6^^,^^7^^]^, and pesticidal^[^^8^^,^^9^^]^ agents. The anticancer activities of vanillin semicarbazone^[^^10^^]^ against Ehrlich Ascites Carcinoma (EAC) have been reported. EAC cells are experimental tumor models used worldwide in cancer research. In 1907, Paul Ehrlich discovered this tumor in the mammary gland of a white mouse, and the tumor was named after him. The present form of EAC cells has been developed by Loewenthal and Jahn^[^^11^^]^ from one of the several carcinoma lines^[^^12^^]^. Khanam et al.^[^^13^^]^ showed that acetone semicarbazone exhibits anticancer activities against EAC cells in Swiss albino mice. Other than Schiff bases, Schiff base complexes with transition metals have also been investigated for their anticancer activities against EAC cells in Swiss albino mice^[^^14^^-^^16^^]^.

In the present investigation, we selected benzophenone semicarbazone (BSC) as the test compound and studied its anticancer activities against EAC cells *in vivo*. Cancer chemotherapy causes myelosuppression and anemia^[^^17^^,^^18^^]^ because of the reduction of both red blood cell (RBC) content and hemoglobin percentage. In support of this anticancer study, hematological parameters have also been studied accordingly.

## Materials and Methods

### Experimental animal

Swiss albino mice of 5 weeks to 7 weeks old, weighing 25 g to 30 g were collected from the International Centre for Diarrhoeal Disease Research, Bangladesh (ICDDR,B) Mohakhali, Dhaka. The mice were kept in iron cages with sawdust and straw bedding that was changed once a week regularly. Standard mouse diet (recommended and prepared by the ICDDR,B) and water were given in adequate amounts. The protocol used in this study for the use of mice as the animal model for research was approved by the University Animal Ethical Committee (27/08/RUBCMB).

### Synthesis of BSC

The procedure for the BSC synthesis was similar to that previously described^[^^19^^]^. The formation and purity of the compound was confirmed by taking melting points and conducting infrared spectral studies. Structure of BSC:


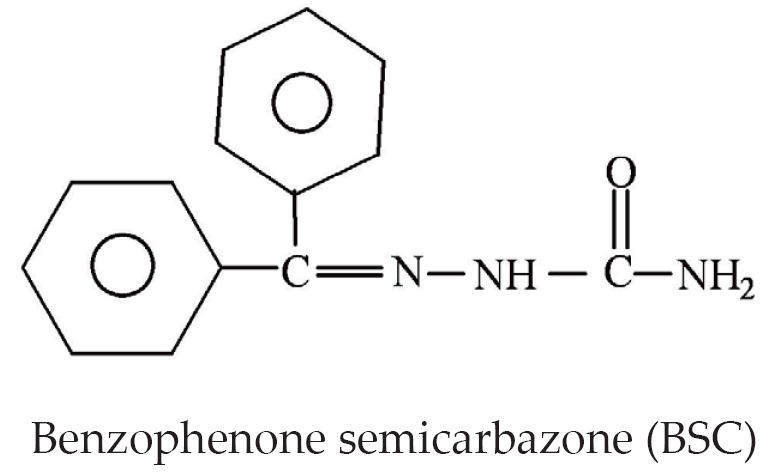


### Tumor cells

Transportable tumor (EAC) cells were used in this experiment. The initial inoculum of the EAC cells was provided by the Indian Institute of Chemical Biology, Kolkata, India. The EAC cells were then propagated in our laboratory biweekly through intraperitoneal (i.p.) injections of 3 × 10^6^ cells (freshly drawn from a donor Swiss albino mouse, bearing 6- to 7-day-old ascites tumor cells).

### Determination of median lethal doses (LD_50_)

The LD_50_ values were estimated by conducting the acute toxicity test, as described elsewhere^[^^20^^]^. The test compound was dissolved in 3% dimethyl sulfoxide, administered intraperitoneally to the different groups with increasing doses. Four animals were placed in each group. Mortality was determined after 24 hours of treatment. The dose at which 50% of the mice survived was considered the LD_50_ value of the compound.

### Cell growth inhibition

Five groups of mice (4 in each group) weighing 25 g to 30 g were prepared for the experiment. A total of 136 × 10^4^ EAC cells were inoculated into each group of mice on day zero. The treatments were initiated after 24 h of tumor inoculation and continued for 5 d. Groups 1 to 3 received the test compound BSC at doses of 5, 15, and 25 mg/kg (i.p.), respectively. Group 4 received bleomycin at a dose of 0.3 mg/kg (i.p.), and Group 5 was used as the control. The mice in each group were sacrificed on day six, and the total intraperitoneal tumor cells were harvested by using normal saline (0.98%). Viable cells were identified using trypan blue and counted using a hemocytometer. The total number of viable cells in every animal of the treated groups was compared with those of the control (untreated EAC bearing mice) group.

### Average tumor weight and mean survival time

Five groups of mice (4 in each group) were used for the experiment. A total of 136 × 10^4^ EAC cells were inoculated in each mouse on day zero. The treatment was initiated after 24 h of tumor cell inoculation and continued for 10 days. The weight changes in each mouse were recorded daily, and the increase in tumor weight was monitored. The host survival was recorded and expressed as the mean of survival time in days. The percent increase in the life span was calculated using the following formula:


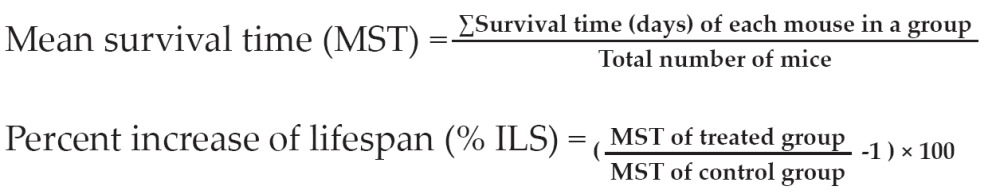


### Hematological parameters in normal and tumor bearing mice

The effects of the test compound on hematological parameters were studied in both normal and tumor-bearing mice^[^^20^^]^. For the tumor-bearing mice, the treatment was initiated after 24 h of tumor transplantation and continued for 10 days. The hematological parameters in the normal mice were studied using a similar method used for the tumor-bearing mice. Blood was drawn out from the tail vein of the mice from each group on days 5, 10, 15, and 25 for such studies.

### Effects of the test compound on normal peritoneal cells

The effects of test compound on the normal peritoneal cells were determined^[^^21^^]^ by counting the total peritoneal cells and the number of macrophages. Four groups of mice (4 in each group) were used in the experiment. Groups 1 to 3 were treated with BSC at doses of 5, 15, and 25 mg/kg (i.p.), respectively, for three consecutive days. Group 4 was used as the untreated control. After 24 h of the last treatment, each animal was injected with 5 mL normal saline (0.98%) into the peritoneal cavity and was sacrificed. The intraperitoneally exuded cells and the number of macrophages were counted with 1% neutral red by using a hemocytometer.

### Statistical analysis

The experimental results were expressed as mean ± SE (standard error).

## Results

In most of the cases, the average values of the repeated experiments were obtained. The lethal dose of BSC was found to be 110 mg/kg for the intraperitoneal treatment of male Swiss albino mice.

The effects of the test compound and bleomycin on EAC cell growth on day six after the tumor transplantation are shown in **Table 1**.

**Table 1:** Effects of the test compound on EAC cell growth *in vivo*.

Treatment Group	Nature of the drug	Dose, mg/kg (i.p.)	No. of EAC cells in mouse on day 6 after tumor cell inoculation	Inhibition of cell growth, %
Control (EAC cell bearing mice)	-	-	(2.517±0.182)×10^7^	-
Bleomycin	Antibiotic	0.3	(0.297±0.012)×10^7***^	88.20
BSC	Synthetic	5	(1.766±0.004)×10^7***^	29.82
		15	(0.962±0.017)×10^7**^	61.80
		25	(0.498±0.007)×10^7***^	80.20

Number of mice in each case was four (*n*=4). Results are shown as mean±SE. Significant values are *P<0.05, **P<0.01, and ***P<0.001.

The treatments with BSC resulted in a cell growth inhibition by 80.20%, 61.78%, and 29.84% for the doses of 25, 15, and 5 mg/kg (i.p.), respectively. The maximum result of 88.20% was expressed by the drug bleomycin at 0.3 mg/kg (i.p.).

All anticancerous drugs showed a significant effect on the survival time of the EAC-bearing mice. The effects of the test compound at different doses are summarized in **Table 2**. The tumor-bearing mice treated with the test compound at different doses showed a significantly increased lifespan. The treatment with BSC increased the life span of EAC cell-bearing mice by 28.12%, 59.87%, and 77.39% for the doses of 5, 15, and 25 mg/kg (i.p.), respectively. Evidently, the survival time increased with increasing dose. Bleomycin at 0.3 mg/kg (i.p.) increased the lifespan by 87.25% compared with the control. The increased percentage in the lifespan of mice at 25 mg/kg (i.p.) of BSC is quite comparable to the effects of the standard anticancer agent bleomycin (0.3 mg/kg).

**Table 2:** Effects of the test compound on the survival time of EAC-bearing mice.

Treatment Group	Nature of the drug	Dose, mg/kg (i.p.)	Mean survival time, day (Mean±SE)	Increase of life span,%
Control (EAC cell bearing mice)	-	-	22.83±0.98	-
Bleomycin	Antibiotic	0.3	42.75±1.12**	87.25
BSC	Synthetic	5	29.25±2.38*	28.12
		15	36.50±1.18**	59.87
		25	40.50±2.42*	77.39

Number of mice in each experiment was four (*n*=4). Results are shown as mean±SE. Significant values are *P<0.05 and **P<0.01.

**Figure 1:**
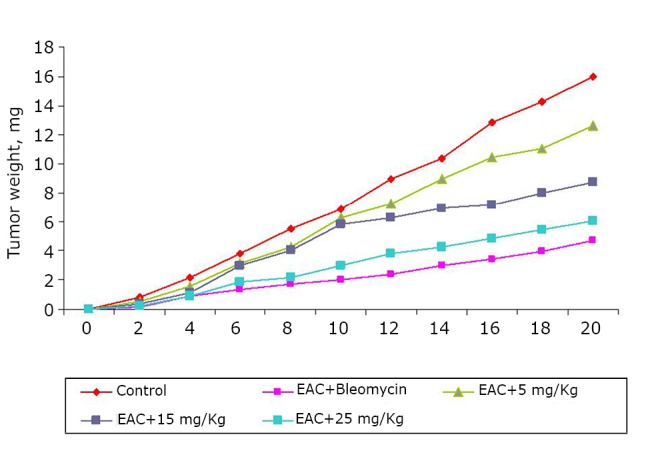
Effect of BSC [at 5, 15, and 25 mg/kg (i.p.)] on average tumor weight. Data are expressed as the mean of the results from 4 mice. Treatment was continued for 10 consecutive days.

The effects of the test compound at different doses and the standard antitumor drug bleomycin (0.3 mg/kg) on the tumor weight are graphed in **Figure 1**. The treatments of the animals previously inoculated with EAC cells with the test compound resulted in tumor growth inhibition.

**Figure 2:**
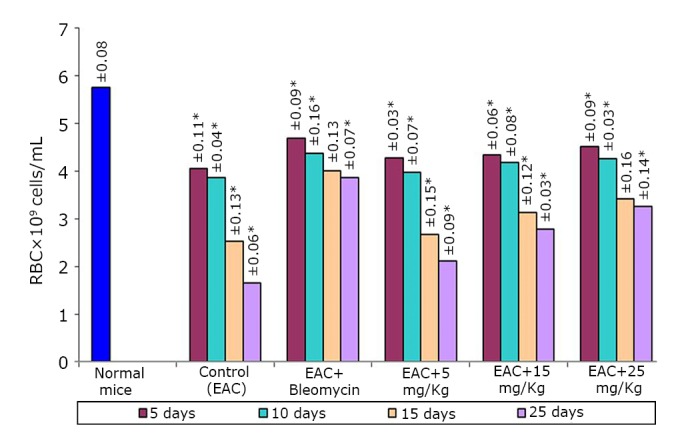
Effect of BSC on RBC of EAC-bearing mice on days 5, 10, 15, and 25. Data are expressed as the mean of the results from 4 mice. Treatment was continued for 10 consecutive days. Significant values are *P<0.05.

**Figure 3:**
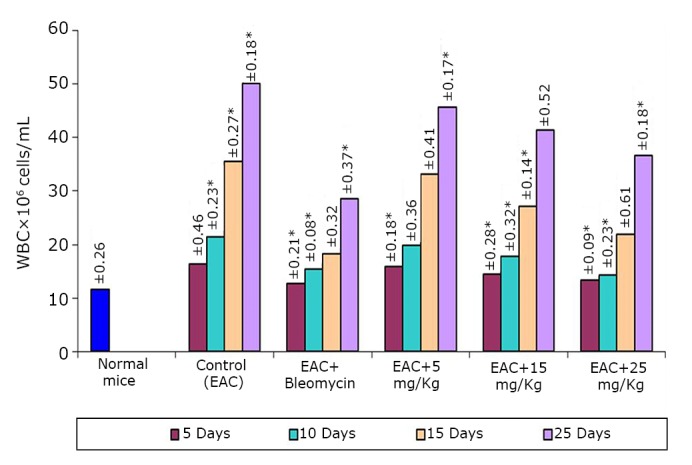
Effect of BSC on WBC of EAC-bearing mice on days 5, 10, 15, and 25. Data are expressed as the mean of the results from 4 mice. Treatment was continued for 10 consecutive days. Significant values are *P<0.05.

**Figure 4:**
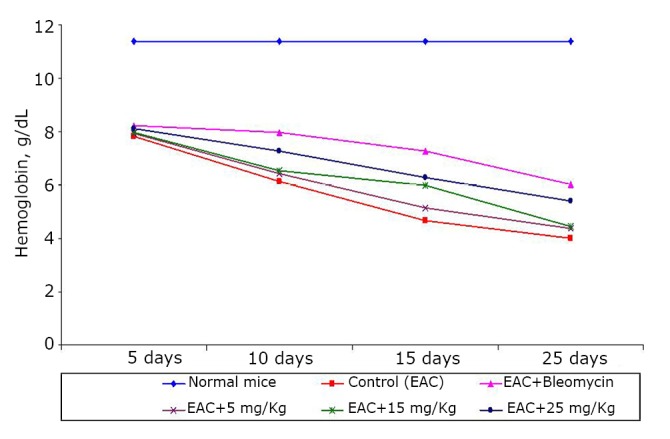
Effect of BSC on the hemoglobin content of EAC-bearing mice on days 5, 10, 15, and 25. Data are expressed as the mean of the results from 4 mice. Treatment was continued for 10 consecutive days.

The effects of the test compound on the hematological parameters of the tumor-bearing mice are shown in **Figures 2****, ****3****, ****4**. The hematological parameters varied from their normal values along with tumor growth. The hemoglobin content and RBC counts decreased, whereas the WBC counts increased after the inoculation of EAC cells. After treatment with the test compound at the previously specified doses, the parameters were restored moderately only at a high BSC dose (25 mg/kg).

**Figure 5:**
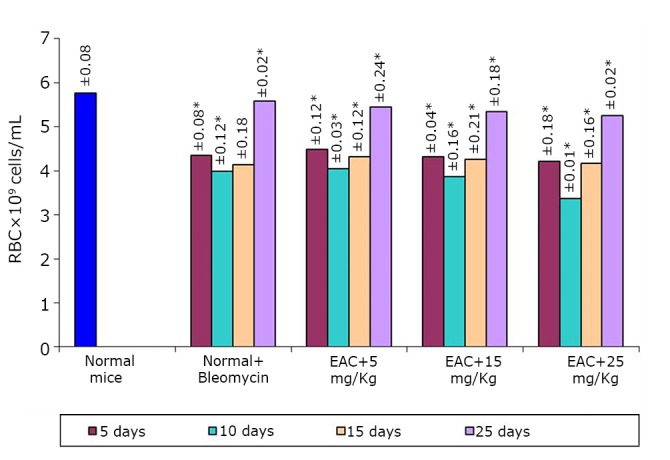
Effect of BSC on RBC of normal mice on days 5, 10, 15, and 25. Data are expressed as the mean of the results from 4 mice. Treatment was continued for 10 consecutive days. Significant values are *P<0.05.

**Figure 6:**
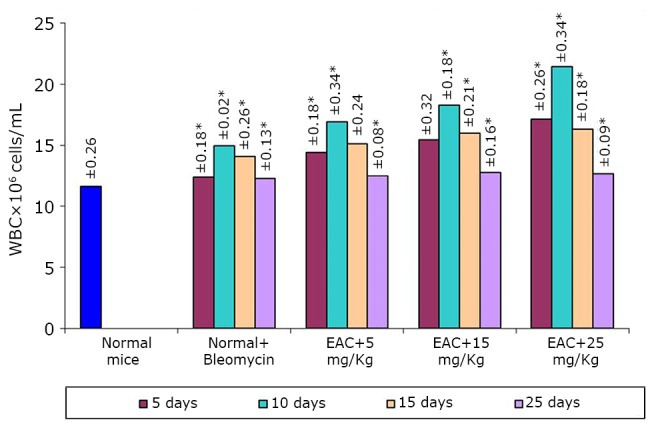
Effect of BSC on WBC of normal mice on days 5, 10, 15, and 25. Data are expressed as the mean of the results from 4 mice. Treatment was continued for 10 consecutive days. Significant values are *P<0.05.

**Figure 7:**
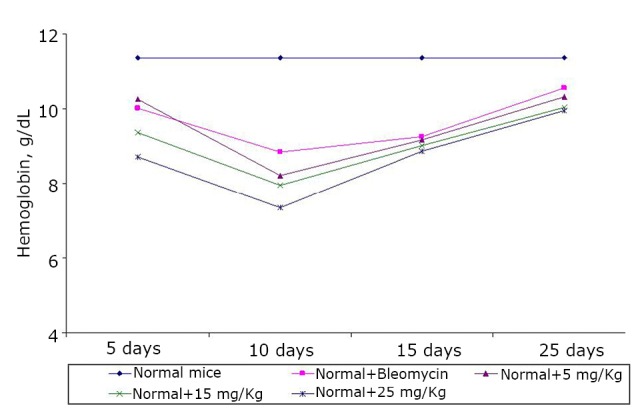
Effect of BSC on the hemoglobin content of normal mice on days 5, 10, 15, and 25. Data are expressed as the mean of the results from 4 mice. Treatment was continued for 10 consecutive days.

The effects of the test compound on the hematological parameters of the normal mice are shown in **Figures 5****, ****6****, ****7**. The test compound showed slight toxicity to the host during the treatment period, but these parameters were almost restored back to normal values after the treatment.

The effects of the test compound on normal peritoneal cells are shown in **Table 3**. The treatment with the test compound at increasing doses resulted in an increase of the normal peritoneal cells and macrophages.

**Table 3:** Effect of the test compound on the enhancement of normal peritoneal cells in mice.

Group	Dose (mg/kg)	Macrophages (cells/mL)	Total peritoneal cells (cells/mL)
Control (untreated)	-	(1.78±0.42)×10^6^	(8.26±0.58)×10^6^
Normal+ BSC	5	(2.12±0.46)×10^6^**	(9.18±0.36)×10^6^*
	15	(2.43±0.28)×10^6^*	(9.67±0.24)×10^6^**
	25	(2.61±0.34)×10^6^	(10.14±0.32)×10^6^*

Number of mice in each group was four (*n*=4). Results are expressed as mean±SE. Significant values are *P<0.05 and **P<0.01.

## Discussion

The potency of BSC as anticancer agent has been judged by measuring the i) inhibition of cell growth, ii) reduction in tumor weight and iii) increase of mean survival time of the EAC-bearing mice^[^^22^^]^. For EAC-bearing mice, the tumor weight has been found to increase rapidly with time. The treatment with the test compound reduced the tumor growth rate. Similar trend has been found in cell growth inhibition ability. The lifespan of the EAC-bearing mice increased remarkably when treated with the test compound. The prolongation of lifespan of cancer bearing mice is a very important and reliable criterion^[^^23^^]^ for judging the potency of any drug as anticancer agent. The positive effect of the compound on EAC-bearing mice has further been verified by monitoring the change in hematological and biological parameters. Both the RBC and hemoglobin content of EAC-bearing mice were found to be decreased gradually with time as found in normal mice. This is probably owing to the deficiency of iron in hemolytic or myclopathic condition^[^^24^^]^. The treatment with the compound has reversed back RBC and hemoglobin contents towards normal. With the growth of tumor, WBC level increased with time. The rise of WBC count of the treated EAC-bearing mice was slower than that in untreated EAC-bearing mice. Parallel hematological experiments have been done with normal mice to evaluate the host toxic effect of the compound. A very slight deterioration in such parameters has been observed during the treatment period. Similar toxic effect observed in bleomycin [0.3 mg/kg (i.p.)] treated mice.

The immunological effect of the test compound in fresh healthy mice have been performed by counting peritoneal macrophages which has provided further support for the potency of the compound as anticancer agent. This compound has notably increased the number of macrophages. This enhancement might produce some cytokinetic products, such as tumor necrosis factor, interleukins, interferons etc. which in turn may be responsible in destroying tumor cells^[^^25^^,^^26^^]^.

Based on the above results, it can be concluded that the test compound showed pronounced activity as an anticancer agent against EAC cells in swiss albino mice. However, the information obtained from the present investigation is insufficient for BSC to be used as a novel anticancer agent in clinical practice. Many more investigation have to be carried out with this compound and its derivatives using various other cancer cell lines and higher animal models.
